# Clinical Cases

**Published:** 1847-12

**Authors:** Benj. F. Wendel

**Affiliations:** New York


Art. II.—*Clinical Cases.* Reported by Benj. F. Wendel, M.D.

I have not before had time to make out a report of the following clinic, although it was delivered by Professor Parker, May 24, 1847, and should not now attempt it did it not seem to me to possess uncommon interest.

Case 1. *Convulsions.*—A child eight months old, of healthy family, some three weeks since had convulsions, and at this time had some twenty a day, according to the mother's account, though last week it had but one, which lasted a quarter of an hour. It vomited before the convulsions came on, and cried much at the same time. It is fed on milk, and sugar and water. Its bowels and water are free; it has not slept well for three weeks; it has never received any injury on the head, and never squinted. Convulsions depend upon some irritation of the nervous system, and the question is, what gave rise to the convulsions three weeks since? did they arise from some irritation in the alimentary canal, which is the most common cause, or from some affection of the brain? During a convulsion, this child does not froth at the mouth, though the hands are clenched; its legs are small, but they are pretty well developed; its head is large, and the vessels large and full; the fontanel is large and elevated, the parietal suture is still open, and on striking we have fluctuation.

Convulsions may arise from some irritation in the alimentary canal, as improper food, dentition, ascarides, etc.; but this child has had good food, dentition is not progressing, and it is not probable that it has ascarides. May the head be in a morbid condition? Most probably it is. The child was restless a day before the convulsions came on, its head was hot, and it vomited prior to the attack, which last is one of the earliest symptoms of disease of the brain; the fontanel is elevated, the sutures open, and by striking fluctuation is perceived; its legs are small, and the head thrown to one side—all these indicating disease of the brain. Its bowels and water are free, which is not generally the case in disease of the brain. Now, if the brain is the primary seat of disease, the chances of recovery are small, and hence our prognosis is unfavorable.

*Treatment.*—Let its head be kept cool, and its extremities warm, friction up and down the spine; and to promote the absorption of the effused fluid, one of the best remedies is hydriodate of potash, of which let the child take one grain four times a day until ten or twelve grains are taken, watching the while if the renal discharge is much increased.

Such a child as this should not be taught to exercise its mind early; precociousness is a bad symptom in children, and they are well enough off if they do not learn their letters at six or seven years old.

Case 2. This is a man complaining of pain in the head, which he has had for two weeks. He had intermittent fever several years ago, and last summer had a pain in the head similar to the present one. He is a full eater, takes a glass of beer once a day; his bowels are regular, and his water is free. The pain is in the temple, under the skull; is of a darting kind, quickly shooting across; affects the left eye and the ears somewhat, though he has no noises in the ears. He was perfectly well during the last winter, and has never suffered any injury of the head, and has not had fever. The antiphlogistic plan has been tried in his case; he has been leeched, cupped, blistered, and purged with salts, without benefit. This is either inflammation involving the bloodvessels, or it is irritation which involves the nerves. There is no inflammation without some irritation, but there may be irritation without inflammation, and a great point in practice is to distinguish between the two. In inflammation the pain is persistent; here it is not. The inefficacy of antiphlogistics leads us to call this a case of irritation, which will not be relieved by such means. This irritation may have arisen in consequence of the intermittent fever which he had years ago.

*Treatment.*—As his tongue is coated, we will clear the way by an emetic and gentle cathartic, and then begin with quinine and morphine, or iron.

Case 3. A peddler, 24 or 25 years old, who had venereal disease eighteen months since, not followed by any blotches or rheumatism, and who never received any injury, was married six months ago, and now complains of pain in the small of the back, and weakness and uneasiness in the limbs after walking. He feels easiest while sitting, and most uneasy while standing; he walks well in the fore part of the day, but in the afternoon has pain in the small of the back, in which region he has had a strengthening plaster, which seemed to do him good; there is no inability to flex the thigh upon the pelvis, nor any tenderness or pain caused by pressure or percussion of the trochanter, to indicate hip disease. In hip disease the patient rises stiff in the morning, while this man rises from bed perfectly well. If there was inflammation, the pain would be increased by walking, while the opposite obtains in this instance. This is a case of debility in the back, and such cases are often seen in boys of sixteen or seventeen years, arising from masturbation, etc. This man admits that he has been in the habit of indulging his sexual propensities with his wife quite freely; his pains did not commence until after his marriage; and these show that his nervous system has been overtasked.

In the treatment, this man is to avoid the exciting cause, apply the cold dash, pouring a bucket of water on the back and hips, keep his bowels free, and take much exercise.

Case 4. An Irishman, about thirty years old, a laboring man, who uses spirits, came in complaining of a steady dull headache, which is worse in the forenoon, especially before breakfast, and of palpitations which render his nights sleepless. His tongue is heavily coated, appetite not good, bowels irregular, bad taste in the mouth on rising in the morning, and continual thirst. The question is, what causes his headache and palpitations? Does the affection depend upon gastric derangement—derangement of the functions of the alimentary canal—or is there disease of the heart? For disease of the semilunar valves we listen between the third and fourth ribs, and at the apex of the heart for disease of the mitral valves. If there is hypertrophy, the heart's apex is nearly if not entirely under the nipple, there is protrusion of that side, and, more of the heart's surface being in contact with the walls of the chest, there is more dullness on percussion. In such cases as this, the pain is more frequently under the pap or to the left of it; but if the trouble is about the heart, the pain is in the left shoulder, and sweeps down the arm of that side.

We must correct this man's digestive apparatus.

Case 5. This is an infant in whom the lymphatic ganglia of the neck are enlarged. Dr. Parker remarked that they would generally go on to suppuration, and recommended a flax-seed poultice.

Case 6. This is a woman who complains of prolapsus of the uterus, and of discharging gravel, which she says were white. As an examination could not be made in this case, Dr. Parker remarked that the phosphatic deposits indicate a depraved state of the system, and were often produced by injuries of the spine; and that in persons of full habits, as the rheumatic, lithic acid and lithate of ammonia occurred.

Case 7. A livery stable keeper, of temperate though full habit, sixty-one years old, has cancer of the lower lip, which has been coming for four years. It commenced in something like a wart, which he was in the habit of pinching off as soon as it came, and for its cure has used various quack medicines; but it has been gradually increasing in size up to this time.

In the treatment of cancer, the less you irritate it the better, for irritating substances always increase the disease. If not disposed to grow, just shield from the air, and if disposed to grow, resort to the knife.

New York, August, 1847.

